# Awareness and knowledge on timing of mother-to-child transmission of HIV among antenatal care attending women in Southern Ethiopia: a cross sectional study

**DOI:** 10.1186/1742-4755-10-66

**Published:** 2013-12-13

**Authors:** Anteneh Asefa, Habtamu Beyene

**Affiliations:** 1 School of Public and Environmental Health, Hawassa University, Hawassa, Ethiopia; 2 SNNPR Health Bureau, Hawassa, Ethiopia

## Abstract

**Background:**

Mother-to-child transmission (MTCT) of HIV infection remains a major public health problem and constitutes the most important cause of HIV infection in children under the age of 15 years old. Awareness on MTCT of HIV and knowledge of its timing usually pose a direct effect on utilization of PMTCT services (mainly HIV testing, infant feeding options and antiretroviral use). The objective of this study is to assess pregnant women’s knowledge on timing of MTCT of HIV in Southern Ethiopia.

**Methods:**

A cross sectional study was conducted in 62 health centers in Southern Ethiopia from February 25 to March 24, 2012. A total of 1325 antenatal care attending women were included in the survey by using a multistage sampling technique. Data were collected using a structured and pre-tested questionnaire. Multiple logistic regression analysis was employed to identify variables associated with women’s knowledge on timing of MTCT of HIV.

**Results:**

All interviewed pregnant women were aware of HIV/AIDS transmission, but only 60.7% were aware of the risk of MTCT. The possibility of MTCT during pregnancy, delivery and breastfeeding was known by 48.4%, 58.6% and 40.7% of the respondents, respectively. The proportion of women who were fully knowledgeable on timing of MTCT was 11.5%. Women’s full knowledge on timing of MTCT was associated with maternal education [AOR = 3.68, 95% CI: 1.49-9.08], and being government employee [AOR = 2.50, 95% CI: 1.23- 5.07]. Whereas, there was a negative association between full knowledge of women on timing of MTCT and no offer of information on MTCT/PMTCT by antenatal care (ANC) service provider [AOR = 0.44, 95% CI: 0.30-0.64], lack of discussion on ANC with male partner [AOR = 0.30, 95% CI: 0.12-0.72], and lack of discussion on HIV/AIDS with male partner [AOR = 0.17, 95% CI: 0.07-0.43].

**Conclusion:**

There was low awareness and knowledge on timing of MTCT of HIV in this study. Hence, strengthening the level of PMTCT services in ANC settings and devising mechanisms to promote involvement of men in PMTCT services is needed.

## Background

Mother-to-child transmission (MTCT) of HIV infection remains a major public health problem and constitutes the most important cause of HIV infection in children less than 15 years old in the globe [1]. In 2012, 260,000 children acquired HIV infection in low and middle income countries and more than 90% of the newly HIV infected children lived in Sub-Saharan Africa, home to 92% of pregnant women living with HIV. Furthermore, only 59% of pregnant women living with HIV in Sub-Saharan Africa received antiretroviral therapy or prophylaxis in the same year [2].

In 2012, the national adult HIV prevalence in Ethiopia was 1.3% and 10% of the 13,008 new HIV infections occurred in children [3]. Among women aged 15–49 years, HIV prevalence was 1.1% in Southern Nations Nationalities and People’s region (SNNPR) in the same year. Moreover, only 9.3% of estimated HIV positive pregnant women in Ethiopia were provided with antiretrovirals [4].

In Ethiopia, only 34% of pregnant women received antenatal care from a skilled provider in the five year period 2006–2010 (27.3% in Southern Nations Nationalities and People’s region), with only 6% increment since 2005 (28%). During the same period, among 226,690 pregnant women who were tested for HIV in the region, 0.5% of them were found to be HIV positive [3,5,6].

Comprehensive knowledge of AIDS is uncommon in Ethiopia. However, 77% of women and 76% of men know that HIV can be transmitted to a baby through breastfeeding, and 44% of women and 53% of men know that the risk of MTCT can be reduced through the use of ARV during pregnancy [5,7].

Limited expansion of PMTCT service and poor integration of with antenatal care (ANC) services are the major gaps and challenges identified in the implementation of HIV prevention in Ethiopia [8]. Hence, strengthening integration of PMTCT services within maternal, newborn and child health is one of the most important strategic focuses for scaling up PMTCT services [4,9].

This study therefore is targeted at identifying possible determinants of knowledge on timing of MTCT of HIV among women attending antenatal care in Southern Ethiopia. Thus findings from this study can be used for evidence based decision making to reduce MTCT of HIV in Sub-Saharan Africa and then to reduce the number of HIV infected children in the world.

## Methods

### Study setting

Considering nearly 90% of the population group of SNNPR are rural dwellers, and among women population (50.48%), 45.3% are in the age group 15–49 years [10], the total number of expected pregnancies in the region in 2011 was 645,550, according to SNNPR Health Bureau’s Report [11]. In 2011/12, there were 520 health facilities which provide PMTCT services in the region [12]. The study was conducted in 62 primary health centers (50 rural health centers and 12 urban health centers) which were selected from nineteen administrative zones of SNNPR between February 25 and March 24, 2012.

### Study design

A cross sectional study which used interviewer administered questionnaire was carried out to assess the awareness and knowledge of antenatal care attending women on timing of MTCT of HIV.

### Study subjects and sampling

Women who attended antenatal care in the selected health centers were the study subjects of this study. Sample size was determined using a single population proportion formula through the following assumptions: confidence interval of 95%, proportion of women who know that HIV can be transmitted from HIV-infected mother to her child by breastfeeding of 77%, according to SNNPR [5], margin of error of 3.3%, a design effect of 2, and non response rate of 10%. Thus the final sample size calculated was 1374. Samples were allocated proportionately using a multistage sampling. First, samples were allocated proportionately to all administrative zones using the expected number of pregnancies in each zone during the year 2009/10. Then, districts were randomly chosen from the zones followed by random selection of health centers providing PMTCT services from the selected districts. Consecutive interview of pregnant women was made until the required samples from every health center were acquired.

### Variables of the study

#### Dependent variables

Women’s awareness on MTCT of HIV and women’s knowledge of timing of MTCT of HIV are categorical dependent variables of the study. Women were considered aware of the possibility of MTCT of HIV when they report HIV could be transmitted from an infected mother to her child. Women’s knowledge on timing of MTCT of HIV was categorized in two groups as followed.

• Some knowledge on timing of MTCT of HIV: when woman reported one or two possible periods of MTCT of HIV (pregnancy, labor/delivery, during breastfeeding).

• Full knowledge on timing of MTCT of HIV: when a woman reported three possible periods of MTCT of HIV (pregnancy, labor/delivery, during breastfeeding).

#### Independent variables

Socio-demographic and economic characteristics (age, marital status, educational status, occupation, male partner involvement and average monthly income), obstetric characteristics (gravidity, gestational age, and number of antenatal care visits), antenatal care service characteristics (whether information on HIV, MTCT/PMTCT and infant feeding was received from service provider or not), and male partner’s characteristics (support during antenatal care and discussion on HIV, and MTCT/PMTCT with a woman during pregnancy) were the independent variables of the study.

### Data collection

Data for this study was collected using a structured questionnaire, pre-tested using 5% of the sample size, originally prepared in English language and then translated to Amharic language.

Data collectors were female nurses recruited and trained to conduct face-to-face interviews with pregnant women. Back translation of Amharic to English language, pre-testing of the tool and close supervision data collection process were undertaken to assure the quality of collected data.

Data were entered into EpiInfo version 3.5.1 and exported to SPSS version 16 to perform descriptive and inferential statistical analysis.

### Statistical analysis

Categorical variables were described through absolute (n) and relative (%) frequencies. The assumption of normality was not attained for continuous variables including age, monthly income, and gestational age after running Kolmogorov–Smirnov test. Hence, medians and interquartile ranges were used to describe those variables. Pearson's chi-square tests and odds ratio (OR) were used to assess the relationship between mothers’ knowledge on MTCT of HIV and other factors. The Cornfield approximation was used for calculating the 95% confidence intervals (CI) for the OR. Multivariate logistic regression was carried out to determine the adjusted effect of each factor on women’s full knowledge on timing of MTCT of HIV. Variables with more than two categories were entered in the model in the form of two “indicator” contrasts comparing each category to the first group as a reference.

A backward stepwise procedure based on the likelihood ratio was used to select the variables included in the final model. The significance for variable removal and entry was set to 0.10 and 0.05 respectively. The Hosmer-Lemeshow test was used to check the goodness-of-fit of the model. Odds ratios and 95% confidence intervals were derived from each variable coefficient in the final model. The significance of each coefficient was tested by the Wald test. Statistical significance was declared at P < 0.05.

### Ethical considerations

Ethical approval was obtained from the Institutional Review Board of SNNPR Health Bureau’s Ethical Review Committee. Written permission was obtained from SNNPR Health Bureau and zonal health departments. Verbal consents were obtained from all interviewed women.

## Results

### Socio-demographic and obstetric characteristics of respondents

A total of 1325 women agreed to participate in the study making the response rate of the study 96.4%. Among the 1325 pregnant women interviewed, 59.4% declared protestant religion 98% were found to be married, 38% did not attend formal education and 55.2% were housewives. Majority (53.2%) of the respondents were in the age group 25–34 years and the median age was 25 years (IQR: 22–29 years). Regarding to the estimated average monthly income, 792 (61.8%) women reported to earn less than 670 Ethiopian Birr per month (Table 1).

**Table 1:** Socio-demographic and economic characteristics of antenatal care attending women, SNNPR, 2012

***Variables***	***Frequency (%)***
**Age (completed years)**	
15-24	540 (40.8)
25-34	705 (53.2)
35-49	80 (6.0)
**Total**	**1325 (100.0)**
**Median (IQR)**	25 (25–75)
**Religion**	
Protestant	787 (59.4)
Muslim	173 (13.1)
Orthodox	314 (23.7)
Catholic	37 (2.8)
Others	14 (1.1)
**Total**	**1325 (100.0)**
**Marital status**	
Single	21 (1.6)
Married	1298 (98.0)
Divorced	3 (0.2)
Widowed	3 (0.2)
**Total**	**1325 (100.0)**
**Educational status**	
Not able to read and write	437 (33.0)
Able to read or write or both	67 (5.1)
Grade 1 – 6	413 (31.2)
Grade 7 – 12	303 (22.9)
College and above	105 (7.9)
**Total**	**1325 (100.0)**
**Occupation**	
Housewife	732 (55.2)
Farmer	174 (13.1)
Pastoralist	6 (0.5)
Merchant	215 (16.2)
Government employee	121 (9.1)
Student	47 (3.5)
Daily laborer	25 (1.9)
Others	5 (0.4)
**Total**	**1325 (100.0)**
**Average monthly income (n = 1282)**	
≤ 670 Ethiopian birr	792 (61.8)
>670 Ethiopian birr	490 (38.2)
**Total**	**1282 (100.0)**

The median (IQR) gestational age of the respondents was 30 (25–75) weeks while the mean gravidity was 2.82 ± 1.75. Nearly two-third (65.7%) of the study subjects were on their 2^nd^ to 4^th^ ANC visit and the mean of antenatal care visits was 2.14 ± 1.08. Majority (69.8%) of respondents reported to have come to the health facility where they sought antenatal care by their own choice (Table 2).

**Table 2:** Obstetrics and ANC service characteristics of antenatal care attending women, SNNPR, 2012

***Variable***		***Frequency (%)***
**Gestational age (in weeks)**	≤ 16 weeks	42 (3.2)
	17-24 weeks	306 (23.1)
	25-35 weeks	756 (57.1)
	≥ 36 weeks	221 (16.7)
	**Total**	**1325 (100.0)**
	**Median (IQR)**	**30 (24–34)**
**Gravidity**	One	360 (27.2)
	Two-four	736 (55.5)
	Five-seven	208 (15.7)
	Eight and above	21 (1.6)
	**Total**	**1325 (100.0)**
**Mothers’ number of ANC visit for their current pregnancy**	First	426 (32.2)
	Second-fourth	871 (65.7)
	Fifth-sixth	22 (1.7)
	Seventh and above	6 (0.5)
	**Total**	**1325 (100.0)**
**Practice of HIV testing during last pregnancy**	Tested	1144(86.3)
	Not tested	181 (13.7)
	**Total**	**1325 (100.0)**
**Had discussion on ANC with male partner during last pregnancy**	Yes	1107 (83.5)
	No	218 (16.5)
	**Total**	**1325 (100.0)**
**Had discussion on HIV/AIDS with male partner during last pregnancy**	Yes	1019 (76.9)
	No	306 (23.1)
	**Total**	**1325 (100.0)**
**Reported level of support respondents got from their male partner during ANC follow up**	Excellent	397 (30.0)
	Very good	721 (54.4)
	Fair	80 (6.0)
	Very poor	37 (2.8)
	No support	90 (6.8)
	**Total**	**1325 (100.0)**

### Women’s practice of HIV testing and male partner involvement in ANC services

The vast majority, 1144 (86.3%) of respondents reported to have undergone HIV testing during their last pregnancy. Similarly, 1107 (83.5%), and 1019 (76.9%) discussed about antenatal care and HIV/AIDS with their current male partner, respectively. Furthermore, majority, 721 (54.4%) of women declared very good support from their current male partners during antenatal care follow up, whereas very poor support and no support at all were declared by 37 (2.8%) and 90 (6.8%) of women, respectively (Table 2).

### Discussion topics covered during ANC

**Figure 1:**
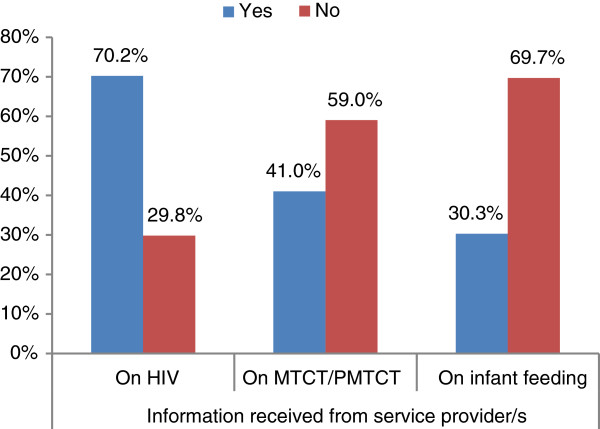
Received Information from ANC service providers, SNNPR, 2012.

Seventy percent of women have had a discussion on HIV with their antenatal care service provider during their visit. However, only 30.3%, and 41.0% of women claimed to have a discussion on infant feeding and MTCT/PMTCT, respectively (Figure 1).

### Pregnant women’s awareness and knowledge on MTCT of HIV and its timing

Though all women were aware of HIV, only 833 (62.9%) of them were aware of the fact that HIV could be transmitted from an infected mother to her child. This awareness was associated with women’s education level (p < 0.001), occupation (p < 0.001), estimated monthly income (p < 0.001), and number of antenatal care visit during the last pregnancy, (p < 0.001).

Regarding knowledge on timing of MTCT of HIV, among who were aware of MTCT of HIV (n = 833), the most known reported timing of MTCT of HIV was delivery (58.6%) followed by pregnancy (48.4%), and breastfeeding (40.7%). There were women who were aware of MTCT of HIV, but did not mention any of the correct timing of MTCT of HIV (14.9%).

In this study, the percentage of respondents who had some knowledge on timing of MTCT of HIV (who reported one or two possible periods of MTCT of HIV) was 39.9% (528) and 11.5% (152) were found to have full knowledge on the timing of MTCT of HIV (who reported three possible periods of MTCT of HIV) (Table 3).

**Table 3:** Awareness and knowledge of antenatal care attending women on timing of MTCT of HIV, SNNPR, 2012

***Variable***	***Frequency (%)***
**Awareness on possibility of MTCT of HIV**	
Aware	833 (62.9)
Not aware	492 (37.1)
**Total**	**1325 (100.0)**
Reported period of MTCT (n = 833)*	
During pregnancy	403 (48.4)
During delivery	488 (58.6)
During breastfeeding	339 (40.7)
Others	51 (6.1)
I don’t know when	124 (14.9)
**Correct timing of MTCT among three possible periods**^**†**^	
None	645 (48.7)
One	282 (21.3)
Two	246 (18.6)
Three	152 (11.5)
**Total**	**1325 (100.0)**

*Multiple response.

†Pregnancy, labor/delivery, and breastfeeding.

### Factors associated with knowledge of women on timing of MTCT of HIV

Full knowledge on timing of MTCT of HIV was associated with socio-demographic and obstetric characteristics of pregnant women. Women who were able to read and write only were found to be more knowledgeable on MTCT of HIV (AOR = 2.97, 95% CI: 1.28 - 6.88). Furthermore, women who had an educational level of grade 7–12, and above were also found to be more knowledgeable on MTCT of HIV, (AOR = 2.52, 95% CI: 1.34 - 4.73) and (AOR = 3.68, 95% CI: 1.49 - 9.08) respectively.

On the other hand, there was a statistically significant difference of knowledge on timing of MTCT among women’s occupation; merchants were less knowledgeable than housewives (AOR = 0.42, 95% CI: 0.21, 0.84). On the contrary, students (AOR = 2.44, 95% CI: 1.09 – 5.47), daily laborers (AOR = 3.05, 95% CI: 1.11 – 8.34), and government employees (AOR = 2.50, 95% CI: 1.23 – 5.07) were more knowledgeable than housewives. However; there was no statistically significant difference of knowledge on timing of MTCT with regards to women’s age, monthly income, gravidity, and gestational week (Table 4).

**Table 4:** Influence of socio-demographic and obstetric factors on pregnant women’s full knowledge level on MTCT of HIV, SNNPR, 2012

**Variables**	**Full knowledge on timing of MTCT**		Crude OR (95% CI)*	Adjusted OR (95% CI)*^†^
	**Yes**	**No**		
**Age in completed years**				
15-24	70	470	1.00	
25-34	73	632	0.78 (0.55, 1.10)	0.87 (0.57, 1.36)
35-49	9	71	0.85 (0.41, 1.78)	1.60 (0.66, 3.91)
**Maximum level of education**				
Not able to read and write	25	412	1.00	
Able to read and write only	9	58	2.56 (1.14, 5.75)	2.97 (1.28, 6.88)**
Grade1-6	29	384	1.25 (0.72, 2.16)	1.31 (0.72, 2.39)
Grade 7-12	50	253	3.26 (1.97, 5.40)	2.52 (1.34, 4.73)**
College and above	39	66	9.74 (5.54, 17.14)	3.68 (1.49, 9.08)**
**Occupation**				
House wife	71	661	1.00	
Farmer	8	166	0.45 (0.21, 0.95)	0.46 (0.20, 1.03)
Pastoralist	0	6	-	-
Merchant	10	205	0.45 (0.23, 0.90)	0.42 (0.21, 0.84)**
Government employee	44	77	5.32 (3.41, 8.29)	2.50 (1.23, 5.07)**
Student	13	34	3.56 (1.80, 7.06)	2.44 (1.09, 5.47)**
Daily laborer	6	19	2.94 (1.14, 7.60)	3.05 (1.11, 8.34)**
Others	0	5	-	-
**Estimated average monthly income**				
< 670 Eth. Birr	66	726	1.00	
≥ 671 Eth. Birr	81	409	2.18 (1.54, 3.08)	1.03 (0.66, 1.61)
**Gravidity**				
One	58	302	1.00	
Two-four	75	661	0.59 (0.50, 0.85)	0.83 (0.53, 1.30)
Five-seven	19	189	0.52 (0.30, 0.91)	1.11 (0.54, 2.29)
Eight and above	0	21	-	-
**Gestational age in weeks**				
≤ 16 weeks	2	40	1.00	
17-24 weeks	33	273	2.42 (0.56, 10.47)	1.69 (0.38, 7.61)
25-35 weeks	87	669	2.60 (0.62, 10.95)	1.96 (0.45, 8.53)
≥ 36 weeks	30	191	3.14 (0.72, 13.68)	2.06 (0.45, 9.32)

^†^All variables were controlled for each other.

*p-value < 0.05, **Statistically significant.

Information received during ANC was also associated with women’s knowledge. Women who did not receive information on MTCT/PMTCT of HIV from ANC service providers during their last pregnancy presented were less knowledgeable on timing of MTCT of HIV (AOR = 0.44, 95% CI: 0.30, 0.64).

Furthermore, discussions on ANC and HIV/AIDS with male partners were also found to have an effect on full knowledge on MTCT of HIV. Thus, respondents who did not have discussion on HIV with their male partner during their last pregnancy were less knowledgeable on timing of MTCT of HIV (AOR = 0.17, 95% CI: 0.07, 0.43) Women who declared very good support from their current male partners during antenatal care were less knowledgeable on timing of MTCT of HIV as compared to those who declared excellent support (AOR = 0.52, 95% CI: 0.35, 0.77) (Table 5).

**Table 5:** Influence of antenatal care service and male partner involvement on pregnant women’s full knowledge level on MTCT of HIV, SNNPR, 2012

***Variables***	**Full knowledge on timing of MTCT**		Crude OR (95% CI)*	Adjusted OR (95% CI*)^**†**^
	**Yes**	**No**		
**ANC visit number of last visit**				
First	44	382	1.00	
Second-fourth	103	768	1.16 (0.80, 1.69)	0.86 (0.56, 1.32)
Fifth-sixth	4	18	1.93 (0.63, 5.96)	1.70 (0.55, 7.07)
Seventh and above	1	5	1.74 (0.20, 15.20)	2.81 (0.26, 28.76)
**Received information on HIV from service provider during last pregnancy**				
Yes	115	815	1.00	
No	37	358	0.73 (0.50, 1.08)	0.83 (0.54, 1.27)
**Received information on infant feeding from service provider during last pregnancy**				
Yes	53	349	1.00	
No	99	824	0.79 (0.55, 13)	0.95 (0.64, 1.41)
**Received information on MTCT/PMTCT from service provider during last pregnancy**				
Yes	95	448	1.00	
No	57	725	0.37 (0.26, 0.53)	0.44 (0.30, 0.64)**
**Had discussion on ANC with male partner during last pregnancy**				
Yes	144	963	1.00	
No	8	210	0.26 (0.12, 0.53)	0.30 (0.12, 0.72)**
**Had discussion on HIV with male partner during last pregnancy**				
Yes	145	874	1.00	
No	7	299	0.14 (0.07, 0.31)	0.17 (0.07, 0.43)**
**Women’s rating of support they got from their male partner during ANC follow up**				
Excellent	78	319	1.00	
Very good	61	660	0.38 (0.27, 0.54)	0.52 (0.35, 0.77)**
Fair	5	75	0.27 (0.11, 0.68)	0.52 (0.19, 1.42)
Very poor	1	36	0.11 (0.02, 0.84)	0.20 (0.03, 1.53)
No support	7	83	0.35 (0.15, 0.78)	0.51 (0.18, 1.46)

†Controlled for age, marital status, educational status, occupation, average monthly income, gravidity and gestational age.

^*^P-value < 0.05, **Statistically significant.

## Discussion

The findings from this study reveal that 100% and 62.9% of women were aware of HIV and possibility of MTCT of HIV from an infected mother to her child respectively. But, only 11.5% of women were fully knowledgeable on the timing of MTCT. Full knowledge on MTCT was associated with women’s educational status and occupation, information received from antenatal care service providers, and discussion with male partners on issues of antenatal care and HIV/AIDS.

In Southern Ethiopia, awareness of ANC attending women on HIV/AIDS was universal, which is consistent with a finding from two similar studies of Nigeria and the Ethiopian Demographic and Health Survey report, 2012 [5,13,14]. Furthermore, the pregnant women’s awareness on MTCT of HIV was lower (62.9%) than similar studies conducted in Arbaminch town in South Ethiopia and Southwestern Uganda, where 80% of women declared that HIV could be transmitted from mother to her child [15,16]. This disparity could be explained by the fact that urban population has more access to information and education than the rural one, which is the focus of this study. Similarly, other studies from Nigeria, Hong Kong, Gondar, Addis Ababa, Tanzania, and Uganda also showed better knowledge of pregnant women on MTCT than our study [13,14,16-21]. This clear difference could be due to the fact that most of these studies were conducted in hospitals and in urban centers, with probable higher access to education, information and knowledge on HIV and its forms of transmission. However, a cross sectional study conducted in a district hospital in Kenya showed a far less level of pregnant women’s awareness on MTCT of HIV, where only 8.9% of mothers knew the possibility of this kind of transmission [22].

Besides, in this study, there was a big gap between awareness on HIV/AIDS and the fact that HIV could be transmitted from an infected mother to her child, which is also supported by a research finding from a teaching hospital in Nigeria [23].

Beyond pregnant women’s awareness on MTCT of HIV, this study has also assessed pregnant women’s knowledge on timing of MTCT of HIV and its association with different variables. The possibility of HIV transmission through breastfeeding was known by 40.7% of women, which is less than the Ethiopian Demographic and Health Survey data where 77.1% of women in SNNPR knew that HIV could be transmitted through breastfeeding [5]. This may be due to regional variation, which may occur within a country. In this study, only 11.5% of pregnant women were fully knowledgeable on timing of MTCT of HIV, which shows a considerable knowledge gap to be addressed.

Maternal education, being a government employee, receiving information on MTCT during antenatal care, having discussions on antenatal care and HIV/AIDS with male partner showed significant association with maternal full knowledge on timing of MTCT of HIV. This finding is in agreement with previous studies in Ethiopia [18,19]. In our study, only 41% of women received information on MTCT/PMTCT during antenatal care, which might have posed a major effect on knowledge on timing of MTCT. This is similar with a finding from South Africa, in which only 35% of HIV positive pregnant women were informed of the possibility of MTCT by their antenatal care counselor [24].

In this study, women’s knowledge on timing of MTCT was positively associated with having a discussion on ANC and HIV/AIDS with their male partner. This is in line with other studies which reported that men involvement in ANC/PMTCT programs to be one of the key success factors which can improve the uptake and outcomes of PMTCT interventions [25,26]. Besides, 6.8% and 2.8% of women claimed to have got no support at all and very poor support from their male partner during antenatal care. This might have happened due to male individual factor barriers, information/knowledge barriers, female individual factors, and relationship dynamics according to a systematic review carried out to identify contributors to low male involvement in ANC/PMTCT services [25].

Though this study encompassed a representative study population from SNNPR, it cannot be generalized to all pregnant women in the region, as there is difference of knowledge among antenatal care attending women and their counterparts.

This is a cross-sectional study and cannot show a cause effect association. Hence, we recommend readers consider a community based study to grasp a clear understanding of level of pregnant women’s knowledge on MTCT of HIV and its timing.

## Conclusion

Antenatal care attending pregnant women’s awareness on MTCT and their knowledge on its timing is still low in Southern Ethiopia. Taking this into account, strengthening the level of PMTCT services in antenatal care settings and devising mechanisms to promote involvement of men in PMTCT services need to be focused on to increase women’s knowledge on MTCT of HIV, in order to reduce its high level in Sub-Saharan Africa.

## Competing interests

The authors declare that they have no competing interests.

## Authors’ contributions

AA conceived the research idea, involved in data collection, analyzed and interpreted the study findings, participated in report writing, and prepared the first draft of the manuscript. HB participated in data collection and report writing, and substantially revised the manuscript for intellectual content. Both authors read and approved the final manuscript.

## References

[B1] UNAIDS Report on the Global AIDS Epidemic 2012 Geneva: Joint United Nations Programme on HIV/AIDS

[B2] UNAIDS Report on the Global AIDS Epidemic 2013 Geneva: Joint United Nations Programme on HIV/AIDS

[B3] HIV/AIDS Estimates and Projections in Ethiopia 2011–2016 Available at http://www.etharc.org/resources/healthstat/hivaids-estimates-and-projections-in-ethiopia-2011-2016. Accessed December 07, 2013

[B4] Federal Ministry of Health Accelerated Plan for Scaling Up Prevention of Mother to Child Transmission (PMTCT) Services in Ethiopia 2011 Addis Ababa: Federal Ministry of Health

[B5] Central Statistical Agency Ethiopian Demographic and Health Survey, 2011 2012 Addis Ababa: Central Statistical Agency

[B6] Central Statistical Agency Ethiopian Demographic and Health Survey, 2005 2006 Addis Ababa: Central statistical Agency

[B7] Federal HIV/AIDS Prevention and Control Office and the World Bank HIV/AIDS in Ethiopia: An Epidemiological Synthesis 2008 Addis Ababa: Federal HIV/AIDS Prevention and Control Office

[B8] Federal HIV/AIDS Prevention and Control Office Report on progress towards implementation of the UN declaration of commitment on HIV/AIDS 2010 Addis Ababa: Federal HIV/AIDS Prevention and Control Office

[B9] World Health Organization PMTCT strategic Vision: 2010–2015 Preventing mother-to-child transmission of HIV to reach the UNGASS and Millennium Development Goals 2007 Geneva: World Health Organization

[B10] Central Statistical Agency 2007 population and housing census of Ethiopia 2012 Addis Ababa: Central Statistical Agency

[B11] Regional Health Bureau SNNP Region overview 2013 Available at: http://www.snnprhb.gov.et/index.php?option=com_content&view=article&id=9&Ite. Accessed February 03, 2013

[B12] Federal Ministry of Health Health and Health-related Indicators Report for EFY 2004 2012 Addis Ababa: Federal Ministry of Health

[B13] Abiodun M Ijaiya M Aboyeji P Awareness and knowledge of mother-to-child transmission of HIV among pregnant women J Natl Med Assoc 2007 99 7 758 763 17668641 PMC2574348

[B14] Umeobika J Ezebialu I Ezenyeaku C Ikeako L Knowledge and perception of mother to child transmission of human immunodeficiency virus among South Eastern Nigerian pregnant women J HIV Hum Reprod 2011 1 1 15 19

[B15] Haddis M Jerene D Awareness of antenatal care clients on mother to child transmission (MTCT) of HIV infection and its prevention in Arbaminch, Ethiopia Eth J Health Dev 2006 20 1 55 57

[B16] Bajunirwe F Muzoora M Barriers to the implementation of programs for the prevention of mother-to-child transmission of HIV: a cross-sectional survey in rural and urban Uganda AIDS Res Ther 2005 2 10 10.1186/1742-6405-2-10 16255776 PMC1277814

[B17] Fung C Loke A HIV/AIDS and risk behaviors in Hong Kong Chinese pregnant women J Adv Nurs 2003 43 3 238 245 10.1046/j.1365-2648.2003.02706.x 12859782

[B18] Tilahun M Degu G Determinant factors of pregnant mothers’ knowledge on mother to child transmission of HIV and its prevention in Gondar town, North West Ethiopia BMC Pregnancy Childbirth 2012 12 73 10.1186/1471-2393-12-73 22838392 PMC3490790

[B19] Teka T Jebessa S Knowledge and attitude towards mother to child transmission of HIV and its prevention among post natal mothers in Tikur Anbessa and Zewditu memorial hospitals, Addis Ababa Eth J Health Dev 2005 19 3 211 218

[B20] Harms G Schulze K Moneta I Baryomunsi C Mbezi P Poggensee G Mother-to-child transmission of HIV and its prevention: awareness and knowledge in Uganda and Tanzania Soc Asp AIDS 2005 2 2 258 266 10.1080/17290376.2005.9724849 17601008

[B21] Bello A Adebimpe W Osundina F Abdulsalam T Perception on prevention of mother-to-child-transmission (PMTCT) of HIV among women of reproductive age group in Osogbo, Southwestern Nigeria Int J Womens Health 2013 5 399 405 23874124 10.2147/IJWH.S45815 PMC3712739

[B22] Omwega A Oguta T Sehmi J Maternal knowledge on mother-to-child transmission of HIV and breastmilk alternatives for HIV positive mothers in Homa Bay District Hospital, Kenya East Afr Med J 2006 83 11 611 618 10.4314/eamj.v83i11.9477 17455450

[B23] Igwegbe A Ilika A Knowledge and perceptions of HIV/AIDS and mother to child transmission among antenatal mothers at Nnamdi Azikiwe University Hospital, Nnewi Niger J Clin Pract 2005 8 2 97 101 16477862

[B24] Chopra M Doherty T Jackson D Ashworth A Preventing HIV transmission to children: quality of counselling of mothers in South Africa Int J of Pediatr 2005 94 3 357 363 10.1111/j.1651-2227.2005.tb03080.x 16028656

[B25] Morfaw F Mbuagbaw L Thabane L Rodrigues C Wunderlich A Nana P Male involvement in prevention programs of mother to child transmission of HIV: a systematic review to identify barriers and facilitators Syst Rev 2013 2 5 10.1186/2046-4053-2-5 23320454 PMC3599633

[B26] Kalembo FW Zgambo M Mulaga AN Yukai D Ahmed NI Association between male partner involvement and the uptake of prevention of mother-to-child transmission of HIV (PMTCT) interventions in Mwanza District, Malawi: a retrospective cohort study PLoS ONE 2013 8 6 1 7 10.1371/journal.pone.0066517 PMC3680434 23776683

